# Reverse RAMP (REVRAMP) pacing: A novel anti tachycardia pacing technique

**DOI:** 10.1016/j.ipej.2021.11.001

**Published:** 2021-11-12

**Authors:** Muzahir Hassan Tayebjee, Robert Bowes, Berthold Stegemann, Arun V. Holden

**Affiliations:** a Department of Cardiology, Leeds General Infirmary, Leeds, UK; b University of Aston, Birmingham, UK; c Faculty of Biological Sciences, University of Leeds, Leeds, UK

**Keywords:** Anti tachycardia pacing, Implantable defibrillator, Painless therapy

## Abstract

Anti-tachycardia pacing (ATP) is frequently used to terminate ventricular tachycardia (VT), however it is not always successful and may accelerate VT requiring defibrillation. REVRAMP is a novel concept of ATP that involves delivering pacing at a faster rate than VT, but instead of abruptly terminating pacing after eight beats, pacing is gradually slowed until VT continues or normal rhythm is restored. In a pilot study we show that REVRAMP can restore normal rhythm, and that if REVRAMP is unsuccessful, VT is not accelerated.

## 1 Introduction

Anti-tachycardia pacing (ATP) is widely used in the painless (i.e. without a defibrillator shock) termination of ventricular tachycardia (VT) in patients with implantable defibrillators [1]. However, it is not always successful and can result in VT acceleration resulting in haemodynamic instability requiring a shock [2].

Reverse ramp (REVRAMP) is a novel concept of delivering ATP. Although the initial pacing cycle length is shorter than the tachycardia, it differs from standard ATP in that the delivery of pacing is longer, and rather than abrupt pacing termination, the pacing cycle length is gradually increased until normal rhythm or VT takes over.

In this pilot study we investigate whether a burst of pacing with a shorter cycle length the tachycardia followed by gradual reduction in the pacing frequency once ventricular overdrive pacing (phase drift) has been confirmed will terminate ventricular tachycardia. This is a refinement of and an addition to current anti-tachycardia pacing technology and could have an important role in reducing the need for terminating VT by a defibrillation shock.

## 2 Methods

All patients who were due to undergo *de novo* defibrillator or cardiac resynchronisation defibrillator implantation were screened, and provided with the patient information sheet if appropriate. Exclusion criteria were: Contraindications to defibrillator testing e.g. severe untreatable coronary disease, intracardiac thrombus, interruption of anticoagulation, participants undergoing box change, device upgrade or revision, inability or unwillingness to provide informed consent. Written informed consent was obtained. This study was approved by the Derby East Midlands Research and Ethics Committee. The study was registered on clinicaltrials.gov NCT03412240.

Following successful secure lead implantation, standard pacing crocodile clips were attached to the pacing electrodes of the right ventricular high voltage lead and connected to an electrophysiology pacing stimulator (Qubic stim, Biotronik, Berlin, Germany). A standard EP recording system (CardioLab, GE healthcare, Chicago, IL, USA) was used to record the ECGs and intracardiac electrograms.

**Fig. 1:**
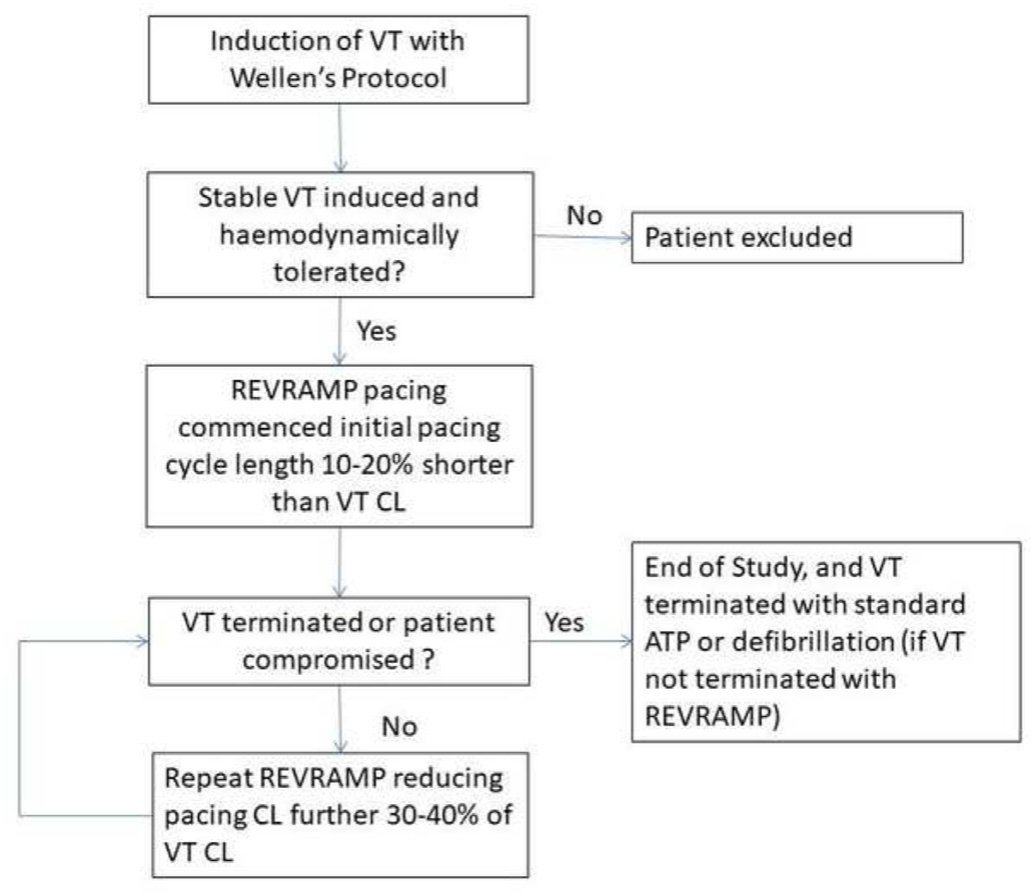
Study Flow diagram (VT, ventricular tachycardia; CL, cycle length; ATP, anti-tachycardia pacing).

Ventricular tachycardia was induced according to the Wellens protocol. If stable VT was induced manually (and sustained for at least 5 seconds) controlled REVRAMP pacing was applied as follows: Burst ventricular pacing (inhibition mode; VVI) was delivered initially at a cycle length 10–20% shorter than the clinical VT. Once phase drift (ventricular overdrive pacing) was been confirmed by showing a change in morphology of the surface ECG or capture of a non-paced intra-ventricular catheter, the pacing cycle length was gradually increased over a period of 5–10s, until either normal rhythm was restored or VT continued. Pacing output was set to maximum (i.e. voltage (10V) and pulse width (1.5 ms)) to ensure myocardial capture. If the first round of REVRAMP cycle was unsuccessful the pacing cycle length was reduced to 20–30% of the tachycardia cycle lengths and the process repeated. The decision to stop anti-tachycardia pacing or protocol testing as well as the maximal pacing rate was left to the operator if termination of VT by REVRAMP was unsuccessful. If REVRAMP was unsuccessful, burst ATP pacing (at much shorter cycle lengths; up to 20 beats could be delivered) or if unsuccessful external defibrillation could be applied (Fig. 1). The primary outcome of the study was VT termination.

In order to minimise the risk of device related infection the pocket was covered with sterile dressings/drapes and standard infection control measures were applied. After 10 minutes, the research protocol was terminated regardless of whether it was possible to induce ventricular tachycardia or test REVRAMP.

## 3 Results

Twelve patients were recruited. All procedures were performed under local anaesthesia with conscious sedation using midazolam or morphine if required. Patients were not sedated prior to induction. Three were excluded (one withdrew, another was unwell on the day and technical problems prevented testing the other). Out of the remaining nine patients stable monomorphic VT was inducible in five patients. All five patients had presented with VT and were therefore receiving a secondary prevention device.

**Fig. 2:**
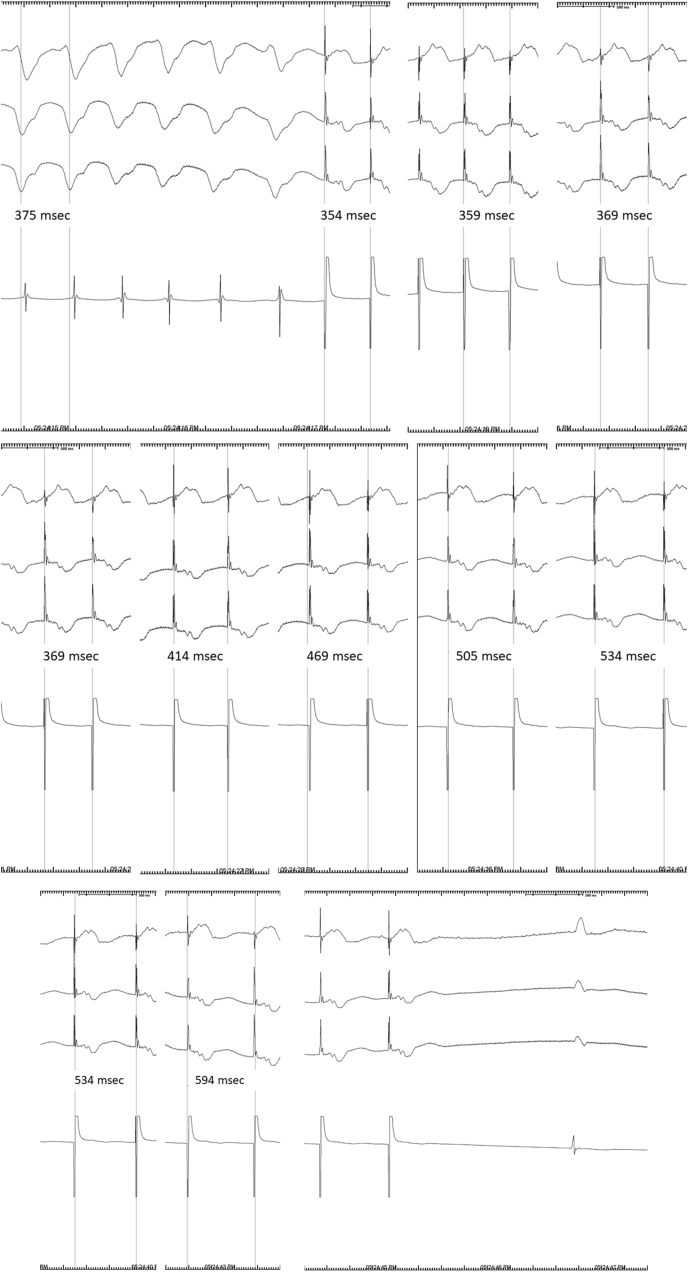
Example of successful REVRAMP pacing. ((a) Induction and initial REVRAMP, (b) continuing of REVRAMP with increase in pacing cycle length, (c) termination of ventricular tachycardia).

**Fig. 3:**
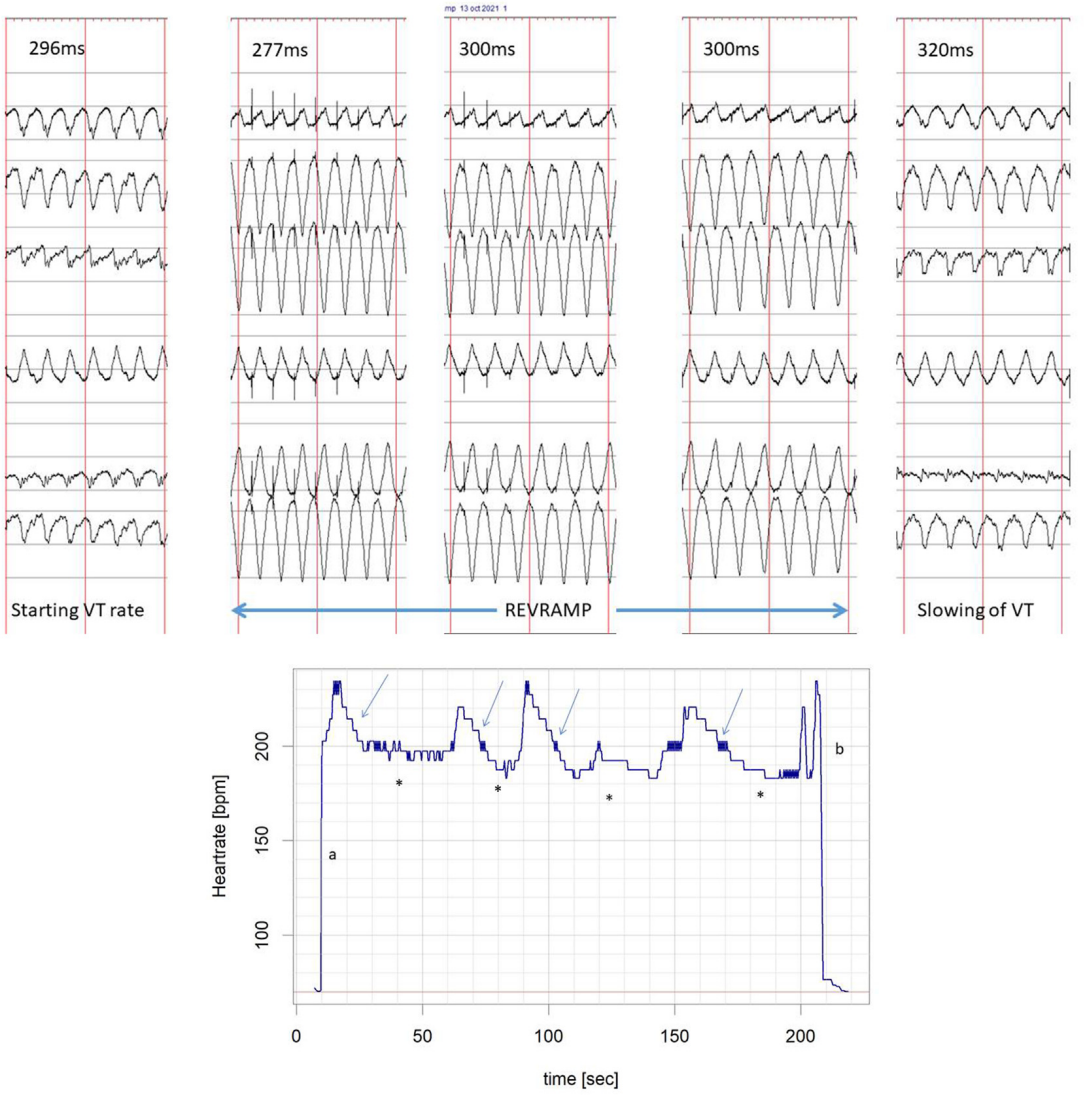
Example of REVRAMP when tachycardia was not terminated but slowed. (a) pacing cyle lengths and ECGs (b) Heart rate change from induction to end of study (a, induction of VT; arrows denote REVRAMP; asterisks show heart rate at end of REVRAMP cycle; b, VT terminated with ATP).

Table 1 shows the baseline characteristics of the patients as well as the VT induction protocol. REVRAMP successfully terminated VT in 2 patients (Fig. 2(a,b,c)). In the remaining three, VT was slowed, but not terminated (Fig. 3(a and b)). No patients suffered an unstable rhythm as a result of acceleration of VT by REVRAMP. In addition, induced VTs in this cohort did not result in haemodynamic instability.

**Table 1:** Patient Demographics and Outcomes of REVRAMP pacing.

Patient	Medical History	Induction sequence (ms) S1,S2,S3,S4	Induced VT CL (ms)	Initial Pacing Rate (ms)	Initial Rate as % of VT CL	Duration (s)	rate/rhythm at end	Successful ATP CL if REVRAMP not successful
1	61 M IHD EF 21%	600,310,250,320	390	360	92	16	420 ms	300 ms
				370	95	20	430 ms	
2	66F IHD EF 31%	400,260,210	296	277	94	10	307 ms	240 ms
				300	101	20	309 ms	
				310	105	10	316 ms	
				300	101	15	320 ms	
				300	101	15	320 ms	
3	69 M NICM EF 40%	480, 200	375	350	93	22	sinus rhythm	n/a
4	51 M IHD EF 43%	600,280,200,340	275	220	80	18	sinus rhythm	n/a
5	77F IHD EF 35%	600,260,300	360	340	94	10	380 ms	260 ms
				290	81	10	380 ms	
				290	81	10	390 ms	

(M, male; F, female; IHD, ischaemic heart disease; NICM, non-ischaemic cardiomyopathy; EF, ejection fraction; VT, ventricular tachycardia; CL, cycle length; ms, milliseconds; s, seconds; REVRAMP, reverse ramp pacing; n/a not applicable).

## 4 Discussion

This is the first report of a novel concept of anti-tachycardia pacing to terminate VT. We document successful VT termination in 2 patients, and no acceleration of the VT but slowing in the patients where REVRAMP did not stop VT.

Sudden termination of anti-tachycardia pacing as is delivered by current defibrillators can lead to a short long interval which could accelerate VT into a much more unstable rhythm requiring defibrillation. In our experience of REVRAMP including what we observed in this cohort, VT was not accelerated but was slowed or stopped by REVRAMP. Therefore more aggressive pacing could be delivered with REVRAMP with a higher chance of VT termination at a much lower risk of VT acceleration. The latter observation is clinically attractive and could possibly be a result of the absence of a short long interval and thus preventing the wave front from recruiting a faster anatomical pathway [3]. The mechanism behind tachycardia slowing could be the induction of functional refractoriness in the VT circuit or a change in the exit site. The latter is less likely as we did not see a change in QRS morphology after pacing.

In this study, the initial pacing rates were chosen and controlled by the operator, and excessively aggressive rates were avoided initially. It remains to be determined what the optimal starting cycle length should be and how long it should be delivered for, but from our observations, pacing does need to be delivered for longer than in standard ATP.

These are initial results of a small observational study. The initial overdrive rate and speed of increase of paced ventricle cycle length had not been standardised, but left to the operator. It was for this reason that elected not to recruit the numbers initially planned. REVRAMP offers advantages over standard ATP, however further work to determine optimal pacing protocols in models [4] is needed (including measures to ensure that defibrillators can detect that ventricular overdrive pacing is successful possibly by using template matching), before testing the technique in a larger trial against standard ATP currently available on defibrillators.

## Funding

An unrestricted research grant was received from Medtronic.

## Declaration of competing interest

MHT has received research funding from Medtronic. BS was previously employed as a principle research scientist with Medtronic.
